# Health Tract, No. 217

**Published:** 1864-09

**Authors:** 


### HUNGER.

THE feeling of discomfort which arises from hunger is referred by the mind to the stomach, although it is only the sensation produced by the wants of the whole body, which make themselves felt there ; every part of the system needs nutriment, but if each individual part suffered with equal intensity, the whole nervous system would be so deranged as either to cause convulsions or such a general disturbance of the machinery of life, that it would not work at all. If the feeling of hunger is allowed to last too long as to the stomach, it loses the power of action and the man dies. It is thus with cough; the cause of cough is in the lungs; there is something there which nature wants away, and which is a hurtful intruder; but the sensation which induces cough is referred to the throat, and trying to repress cough, either by the force of the will or anodyne medicines, is as unphilosophical as useless, as fatal in the end as trying to repress hunger, without removing the cause of it, that is, without supplying the needed nutriment.

When a person is really hungry, the system has sent certain materials to the stomach from which the liquid is manufactured which is necessary to the digestion of the food, and as soon as the food is swallowed, this liquid begins to act upon it, and the sense of hunger gradually subsides. But when the system has not used up all the nourishment which the previous meal had supplied, there is no sensation of hunger, there is no call for food, there is no appetite, which means a "seeking for," there is no need for eating, for nature never tells a lie; no liquid has been prepared which induces the sensation of hunger, hence nothing to digest the food if conveyed into the stomach, and if food is eaten under such circumstances, there being nothing in the stomach to take hold of it and convert it into nutriment, it remains for hours causing discomfort, heaviness, and even convulsions. At length it begins to decompose, to "rot;" wind is generated, and the most noisome eructations and other gaseous discharges are made. Sedentary persons do not "get over" these wicked impositions on nature for days and weeks sometimes. This is the philosophy of the folly, the brutality, the criminal waste, and the sin of "eating without an appetite."

